# Color Change and Color Stability of White Spot Lesions Treated with Resin Infiltration, Microabrasion, or Nano-Hydroxyapatite Remineralization: An In Vitro Study

**DOI:** 10.3390/dj13030112

**Published:** 2025-03-03

**Authors:** Nina Novozhilova, Anastasia Mun, Maria Polyakova, Anna Mikheikina, Alexandr Zaytsev, Ksenia Babina

**Affiliations:** 1 Department of Therapeutic Dentistry, I.M. Sechenov First Moscow State Medical University (Sechenov University), 119991 Moscow, Russia; novozhilova_n_e@staff.sechenov.ru (N.N.); mun_a_s@student.sechenov.ru (A.M.); polyakova_m_a_1@staff.sechenov.ru (M.P.); mikheykina_a_m@staff.sechenov.ru (A.M.); 2 Institute of Linguistics and Intercultural Communication, I.M. Sechenov First Moscow State Medical University (Sechenov University), 119991 Moscow, Russia; zaytsev_a_b@staff.sechenov.ru

**Keywords:** coffee, color stability, dental caries, hydroxyapatite, enamel microabrasion, masking effect, resins, synthetic, tooth remineralization, white spot lesion

## Abstract

**Background**: We compared the camouflage effect of three white spot lesion (WSL) treatments (infiltration, nano-hydroxyapatite (nHAP) remineralization, and microabrasion) and color stability of the treated surfaces. **Methods**: Fifty sound extracted teeth were used in the study. WSLs were created on 40 buccal dento-enamel specimens through the use of acidic methylcellulose gel. These specimens were randomly assigned to treatment groups (*n* = 10 per group): negative controls, nano-hydroxyapatite (nHAP), resin infiltration, and microabrasion. After the treatment, all 50 specimens were immersed in coffee for 7 days. Color measurements were performed four times: at baseline (T0), after the demineralization procedure (T1), after the treatments (T2), and after immersion in coffee (T3). **Results**: No restoration of the initial enamel color was observed in any of the groups. The ICON and MA groups exhibited the highest masking effect, with the mean ΔE_T0-T2_ = 7.46, although the differences among the study groups were insignificant. All three treatments increased the resistance of WSLs to discoloration in coffee compared to the negative control group; however, infiltration (∆E_T2-T3_ = 4.13) and microabrasion (∆E_T2-T3_ = 3.49) showed a better color stability tendency than nHAP remineralization (∆E_T2-T3_ = 7.26). **Conclusions**: Despite its well-known remineralizing and desensitizing effects, nHAP showed the least masking effect for WSLs and lower color stability compared to resin infiltration and microabrasion. However, none of the methods allowed for complete restoration of the original color. After the discoloration procedure, the color changes in the white spots treated with microabrasion and infiltration were comparable to those of the sound enamel.

## 1. Introduction

Dental caries remain the most prevalent oral condition in childhood and adulthood that may adversely affect quality of life [1,2,3,4]. Despite a large amount of research in the field of caries management and prevention, the prevalence of this disease has not changed considerably in recent decades [5]. Dental caries is a multifactorial condition caused by mineral loss due to an imbalance between the dynamic processes of mineralization and demineralization of hard tooth tissues [6,7,8]. When demineralizing episodes prevail, the earliest clinical evidence of enamel demineralization, a white spot lesion (WSL), may develop [7,8,9]. The dyschromia of an early caries lesion can be explained by the differences in refractive indices between the healthy enamel and the demineralized area [10,11]. This results in a stronger scattering of light within the lesion, giving rise to its whitish, opaque, and chalky appearance [7,12]. Incipient enamel caries are smooth on probing, as the surface layer is relatively unaffected at this stage [9,11]. However, if untreated, this layer may be destroyed, leading to enamel cavitation [7]. At the same time, early non-cavitated caries may be reversed or arrested [8].

White spot caries management is presently focused on targeted conservative approaches to arrest or reverse caries progression, as well as to mask the initial lesion [10,12,13,14,15]. Within this concept, the treatment of WSLs primarily includes non-invasive options such as remineralization and minimally invasive options such as resin infiltration or microabrasion [14].

Remineralization can be achieved through increasing the mineral content in the demineralized enamel to form more stable compounds that are less soluble in acids [16,17,18]. The use of fluoride-based agents is considered to be the gold standard in the prevention and management of early caries lesions [7,10]. Although these agents are effective for superficial remineralization, the body of the lesion is left unaltered [19]. As a result, enamel appearance remains impaired [10,12]; thus, fluorides do not solve the esthetic problem which is of key importance in anterior teeth [11]. Moreover, surface hypermineralization formed by the use of high concentrations of fluoride may arrest deeper remineralization [20,21] and increase the risk of dark staining, which might further compromise the esthetic treatment result [11,21]. To address the aforementioned limitations, novel approaches have been developed, with biomimetic nanohydroxyapatite (nHAP) being the most promising [19,22,23,24,25,26,27]. There is growing evidence of greater remineralizing efficacy of nHAP compared to fluoride [19,28,29]. Nano-HA has a great affinity to the enamel as it is chemically similar to the apatite of the enamel crystals [30,31]. Due to its small size, it can easily penetrate into the pores of the enamel rods, thus providing restoration of WSLs [28,32,33]. It has been shown recently that nHAP is effective not only in remineralization, but also in masking WSLs [14,34].

Enamel microabrasion is another treatment option for prompt improvement in the appearance of the incipient enamel lesion [10,12,21]. The key mechanism of enamel microabrasion is the chemical erosion and mechanical abrasion of the outer defective enamel layer [15,21,35]. During the procedure, application of a slurry of hydrochloric acid (HCl) and silica particles results in mild abrasion of the superficial demineralized enamel and exposure of a highly polished surface layer resulting in an improvement in lesion appearance [14,21]. It should be noted that this method is mainly limited for superficial lesions [11].

Recently, resin infiltration, a minimally invasive treatment approach, was introduced, in which the WSL is infiltrated using a low-viscosity light-cured methacrylate resin [36,37]. In this technique, hydrochloric acid is used to remove the surface layer of the decalcified enamel to create access to the body of the lesion, thus facilitating resin penetration [10,38]. Studies have shown that resin infiltration has the ability to seal the caries lesion and prevent its progression [39,40,41]. Moreover, the resin refractive index is similar to that of the enamel; therefore, infiltration provides a camouflage effect and masks the opacities caused by demineralization [7,11,37,38].

Apart from immediate masking ability, resistance against extrinsic discoloration affects the long-term esthetic outcome of WSL treatment [36,39,42]. A number of studies have assessed the effectiveness of different methods of treating WSLs [7,15,32,33,38,43], but there is still no consensus on the topic. To the best of our knowledge, only a few studies compared the immediate masking effect and color stability of WSLs treated with resin infiltration, microabrasion, or nHAP. Therefore, the aim of our study was to compare the camouflage effect of three WSL treatment approaches (infiltration, nHAP remineralization, and microabrasion) and to assess the resistance of the treated surfaces against extrinsic discoloration.

## 2. Materials and Methods

### 2.1. Specimen Preparation

Caries-free human third molars and premolars without cracks, stains, or developmental abnormalities on the buccal surfaces were used in this study. The teeth were collected from the consenting patients visiting our Dental Center (Sechenov University, Moscow, Russia) in the context of a treatment plan. The Ethics Committee of Sechenov University approved the trial protocol (No. 0417, 17 April 2017).

The specimens were rinsed with tap water and scaled to remove organic and inorganic debris. Then, the teeth were disinfected in a 0.1% thymol solution (DR Thym™, Hatley, QB, Canada) for 48 h. The teeth were stored in distilled water in a refrigerator (4 °C) before the start of the experiment [15].

Each tooth was sectioned mesio-distally using a diamond bur in a high-speed handpiece (NSK, Kanuma, Togichi, Japan) to acquire buccal dento-enamel specimens 4 mm thick as measured with an electronic calliper (Mechanic 150 Pro, ADA Instruments, Jiangsu, China). The soundness of the specimens was assessed after preparation using an operating microscope (OPMI PROergo, Carl Zeiss Meditec AG, Oberkohen, Germany), and those with scratches and cracks were excluded.

A total of 40 specimens underwent demineralization, and 10 specimens were not demineralized (the sound enamel group). Circular plastic stickers 5 mm in diameter were glued to the center of the enamel surface of the specimens. The remaining surface was coated with a translucent acid-resistant nail varnish (Revlon, Paris, France) twice [43]. After the removal of the stickers, the enamel surface was polished with fine grit (20 μm) and extra-fine grit (10 μm) aluminum oxide polishing disks (OptiDisk, Kerr, Bioggio, Switzerland). Then, all specimens (*n* = 50) were stored in distilled water for 24 h to avoid color alterations due to dehydration, and baseline color measurement was performed (T0).

Dental plaque was simulated through the use of acidic methylcellulose gel [15]. The gel was prepared by mixing of 8 g of carboxymethylcellulose gel (Xi’an Geekee Biotech Co., Ltd., Shaanxi, China) with 100 mL of 0.1 M lactic acid (Medpack LLC, Kirov, Russia). The pH was adjusted to the level of 4.6 using a 45% KOH solution (Belaruskali JSC, Soligorsk, Belarus). The specimens were stored in covered vials containing demineralizing gel in an incubator (DSI-060D, Digisystem Laboratory Instruments, Inc., New Taipei City, Taiwan) at 37 °C for 14 days. Throughout the incubation period, the pH was maintained at 4.6, as confirmed by a digital pH meter (Milwaukee pH56 PRO, Rocky Mount, NC, USA). The sound enamel specimens were stored in carboxymethylcellulose gel without lactic acid in similar conditions. Then, color measurement of the demineralized and sound enamel samples was performed (T1).

### 2.2. Treatment of WSLs

The specimens (*n* = 50) were randomly assigned to the groups.

- Positive control (sound enamel): The specimens were not demineralized and remained untreated (*n* = 10).
- Negative control: After demineralization, the specimens remained untreated (*n* = 10).
- nHAP: A small amount of APAPRO paste (SANGI Co., Ltd., Tokyo, Japan) was applied to each demineralized specimen using a rubber cup and low-speed handpiece (500–750 rpm) for 30 s according to the manufacturer’s instructions [44]. After that, the specimens were rinsed with water (*n* = 10).
- MA: A small amount of abrasive slurry Opalustre™ (Ultradent Products, Inc., South Jordan, UT, USA) was applied to each demineralized specimen using a rubber cup (Opal Prophy Cups, Ultradent Products, Inc., South Jordan, UT, USA) and low-speed handpiece (5000 rpm) for 60 s [14]. After that, the specimens were rinsed with water (*n* = 10).
- ICON: 15% HCl (Icon Etch, DMG, Hamburg, Germany) was applied to each demineralized specimen for 120 s, rinsed off for 30 s, and air-dried using an air–water pistol. Then, 99% ethanol (Icon Dry, DMG, Hamburg, Germany) was applied for 30 s. Next, resin infiltrant (Icon Infiltrant, DMG, Hamburg, Germany) was applied, left for 3 min to allow the resin to penetrate into the lesion, and polymerized for 40 s (Demi Plus LED light-curing system, Kerr, Orange, CA, USA). Then, a second 1 min application was performed to compensate for polymerization shrinkage. The surface was polished with rubber cups (Swiss Flex, Coltene Whaledent, Altstatten, Switzerland) (*n* = 10) [40].

After the treatments, the specimens were rinsed with distilled water and the nail varnish was removed with acetone. Then, the specimens were stored in distilled water for 24 h to prevent dehydration, and color changes after different treatments were assessed (T2).

### 2.3. Discoloration Procedure

After the T2 measurements, all specimens were immersed in staining solution. Freshly brewed coffee (Ethiopia Yirgacheffe dark-roasted beans) from a coffee machine was used to imitate dietary staining [45]. Each specimen was immersed in a plastic box containing 30 mL of coffee at room temperature over a 7-day period. Then, the specimens were rinsed and stored in distilled water for 24 h, and color changes were assessed (T3).

### 2.4. Color Measurements

Color and spectral distributions were assessed according to the Commission International de l’Eclariage (CIE) L*a*b* system (CIE Colorimetry Publication, 1986).

Each color measurement was performed using a CR20 colorimeter (CHN Spec Technology Co., Ltd., Zhejiang, China) with an aperture size of 4 mm at a right angle after calibration using a white calibration cap included in the kit. All measurements were performed by the same operator. In order to reduce the effect of external light, all measurements were carried out at midday in the same place every time. A gray background was used as it was found to produce a lower influence on the color. The following parameters were registered: index *L*—brightness axis that varies from 0 (black) to 100 (white); index *a*—red-green color axis; and index *b*—blue-yellow color axis [46].

Color measurements were performed four times: at baseline (T0), after the demineralization procedure (T1), after the treatments (T2), and after immersion in coffee (T3).

The total color change (ΔE) between the study timepoints was calculated according to the following formula:

(1) $$\Delta E=\sqrt{{\left(L_{1}^{*}-L_{2}^{*}\right)}^{2}+{\left(a_{1}^{*}-a_{2}^{*}\right)}^{2}+{\left(b_{1}^{*}-b_{2}^{*}\right)}^{2}}$$

where L*_1_, a*_1_, and b*_1_ indicate initial readings and L*_2_, a*_2_, and b*_2_ indicate final readings.

The colorimeter was calibrated before each measurement. Color measurements were performed immediately after the removal from distilled water and drying with a filter paper. Each color measurement was performed thrice, and a mean value was calculated for each specimen.

### 2.5. Statistical Analysis

The data were analyzed with R language (R Development Core Team, Columbia university, New York, NY, USA), version 4.2.3, 2023-03-15 in RStudio software (Posit Software, PBC, Boston, MA, USA), version 2023.03.0+386. Shapiro–Wilk’s test was used to check the normality of the data. To find the significant differences within the groups for L*, a*, b* parameters at different time intervals, a repeated measures ANOVA was used, followed by paired Student’s *t*-test. Between the groups, a post hoc Tukey test was used for the intergroup comparisons. One-way ANOVA with a post hoc Tukey test was applied for ∆E values.

Sample size was calculated based on the results of a study by Hammad et al. [14] for the ∆E values (difference between the baseline color and the color immediately after the treatments). The mean values for the four study groups were 15.79, 11.99, 10.96, and 3.00; the standard deviation was 1.655. The power was set as 80% and the *p*-value was set as 0.0125 (to account for multiple comparisons). The required sample size per group was 2 samples per group, but it was decided to increase it to 10 per group, as this was the sample size in the majority of similar studies.

## 3. Results

**Figure 1:**
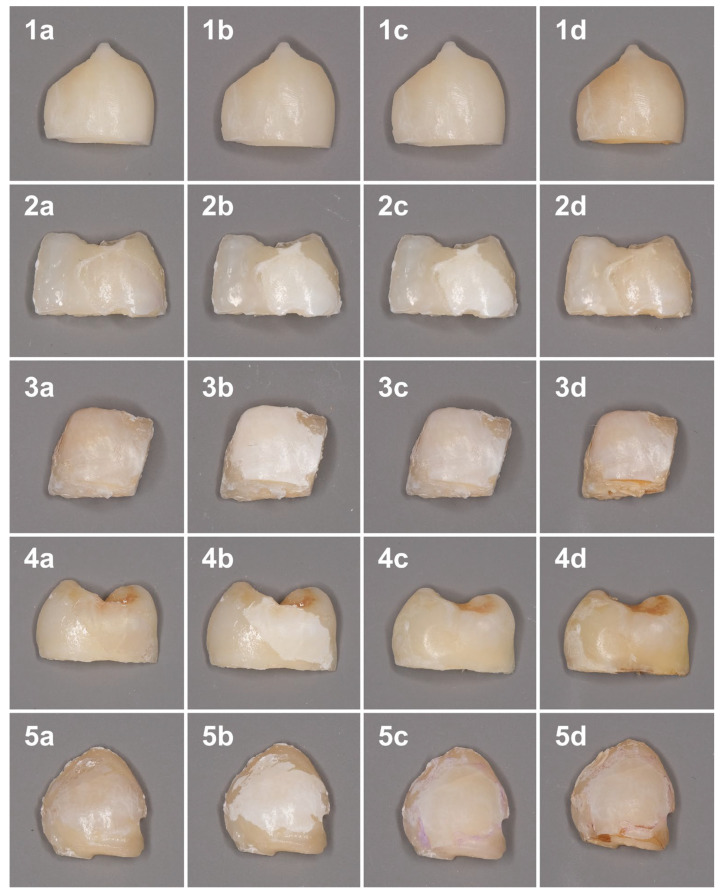
Color changes of the samples at different time points. 1—Sound enamel; 2—Control; 3—nHAP; 4—ICON; 5—MA. (**a**) Baseline; (**b**) After demineralization procedure; (**c**) After treatment; (**d**) After discoloration procedure.

The color changes at different timepoints are shown in Figure 1.

According to repeated-measures ANOVA, the “timepoint”, the “group”, and the interaction of these factors had a significant impact on the L* and b* values, although only the “timepoint” and the interaction factors had a significant impact on the a* value. Color lightness values (L*) did not differ significantly among the study groups at baseline (T0) and after the demineralization procedure (T1) and ranged between 69.36 and 74.0 and between 79.53 and 83.03 in demineralized specimens, respectively (Table 1). After demineralization, all groups demonstrated a significant increase in the L* parameter at T1 compared with T0. No differences were observed in color chromaticity (a* and b*) among the groups at T0 (Table 2 and Table 3). A slight decrease in the mean a* values was observed at T1 compared with T0. The mean b* values in all groups at T0 were positive and ranged between 5.10 and 7.05; after demineralization, the b* values became negative, indicating a shift towards the blue color. In the positive control group that was not demineralized, no changes in either parameter were registered. At T1, the L* and b* values differed significantly between the sound enamel and demineralized specimens, while there were no significant differences in the a* parameter.

After the treatments, the L* value decreased in the MA and ICON groups (*p* < 0.001 and *p* = 0.00949, respectively). The values were 67.08 ± 4.43 in the resin infiltration group and 70.34 ± 1.13 in the microabrasion group (*p* = 0.8059788). No significant differences in this parameter were found between T1 and T2 in the positive control (sound enamel), negative control, and nHAP groups (*p* = 0.877 *p* = 0.368, and *p* = 0.905, respectively). At T2, the L* and b* values in the MA and ICON groups did not differ significantly from those of sound enamel. Moreover, the b* value in the MA and ICON groups became positive, suggesting an increase in the intensity of the yellow color after the treatments. There were no significant changes observed in the a* values in all groups at T2 compared with T1 except for the MA group, in which this parameter slightly increased.

Immersion in coffee resulted in a significant decrease in lightness (L*) in the negative control group (the L* value comprised 75,23); at the same time, the specimens exposed to different treatments and sound enamel (positive control) demonstrated no significant lightness change. At T3, the L* values in the MA and ICON groups did not differ significantly from those of the positive control (*p* = 0.944 and *p* = 0.792, respectively). After staining, the a* value increased in the control group (*p* = 0.0178), indicating a change in color chromaticity from green to red; however, the sound enamel, MA, and ICON groups showed no significant changes in this parameter (*p* = 0.403, *p* = 0.117, and *p* = 0.203, respectively).

According to one-way ANOVA, the “group” factor had a significant impact on the ∆E values except for the ∆E_T2-T3_ (*p* = 0.0572). The color change parameter after the demineralization procedure (ΔE_T0-T1_) did not differ significantly among the control group (10.23 ± 1.31), the microabrasion group (11.94 ± 4.08), the nHAP group (11.24 ± 2.05), and the Icon group (13.47 ± 3.94) (Table 4). After treatment of the demineralized specimens, the color of WSLs considerably improved in the ICON and MA groups; ΔE_T1-T2_ comprised 20.98 and 14.40, respectively (*p =* 0.0005838). There were no significant differences between the nHAP group and negative controls in ΔE_T1-T2_ (*p* = 0.4405020). The color of the treated specimens was closer to the baseline in the ICON and MA groups (ΔE_T0-T2_ comprised 7.46 ± 4.53 and 7.46 ± 2.48, respectively). After the discoloration procedure, the smallest changes in ΔE parameter (T_2_-T_3_) were found in the MA and ICON treatment groups. These values were close to the values demonstrated by the sound enamel (positive control) immersed in coffee. Despite a great deal of variation among the ΔE_T2-T3_ values of the groups, the differences did not reach the level of statistical significance. Regarding ΔE_T0-T3_, the ICON group showed the most prominent changes which differed significantly from the positive control and negative control groups.

## 4. Discussion

In the present study, we compared the camouflage effect of three WSL treatments (infiltration, nHAP remineralization, and microabrasion) and the resistance of the treated surfaces against extrinsic discoloration. The highest masking ability was achieved via infiltration and microabrasion. However, none of the methods allowed for complete restoration of the original color. After the discoloration procedure, the color changes in the white spots treated with microabrasion and infiltration were comparable to those of the sound enamel.

The masking effect of microabrasion and infiltration in the management of WSLs is well-documented [7,10,12,21,35,38,47,48,49]. However, there is still no consensus on the optimal treatment approach; moreover, with the introduction of new biomimetic materials, it is important to investigate their remineralizing and camouflage effects in order to promote understanding of possible long-term esthetic outcomes. Nano-HAP has been widely recognized in dentistry for its remineralizing and desensitizing potential [24,26,30]. The synthetic HAP is chemically similar to the apatite constituting human enamel crystals [19]; it has been found to exhibit extreme affinity to enamel and even greater bioactivity than a biological apatite [28,29]. Nanosized HAP particles can penetrate the porous demineralized enamel and enrich it with calcium and phosphate ions, thus providing remineralization of the incipient caries lesions [28,30,33]. Moreover, nHAP was shown to improve the esthetic appearance of WSLs [14].

In the present study, white spot lesions were artificially induced using a method described in previous studies [15,50]. Demineralization of the enamel resulted in lightness (L*) increase in all samples. Chromaticity changes included a decrease in the b* parameter to negative values, indicating a shift towards blue color and a slight decrease in the a* parameter. Similar changes after enamel lesion formation were reported in a study by Yetkiner et al. [47].

We found that, after WSL treatment, both infiltration and microabrasion provided a considerable masking effect. The L* values in these groups did not differ significantly from the baseline levels and from the sound enamel values. The differences in the b* values at T2 did not differ significantly from the baseline and sound enamel values only in the ICON group. A considerable increase in the a* parameter was found in the MA group, indicating a shift towards red color. It may be hypothesized that microabrasion slightly reduced enamel thickness, thus making the underlying dentin more visible. No restoration of the initial enamel color was observed in any of the groups. The ICON and MA groups exhibited the highest masking effect, with the mean ΔE_T0-T2_ = 7.46, although the differences among the study groups were insignificant. Regarding the clinical relevance of these results, there is still no consensus on the threshold of ∆E appreciated by the human eye. Color differences are considered imperceptible for a value less than 1 [51], while ∆E between 1 and 2 is frequently detected by skilled observers and ∆E more than 2 can be noticed by all observers [52]. All in all, most color studies used a threshold of <3.3 for a clinically acceptable result [46,53,54]. Therefore, although the ∆E values of WSLs after infiltration and microabrasion found in the present study were smaller than in the nHAP and negative control groups, they were still higher than the aforementioned threshold.

To the best of our knowledge, only the study by Hammad et al. compared the masking effect of the same WSL treatments [14]. In their study, the resin infiltration group showed the lowest mean of color differences (ΔE = 3.0) between non-demineralized specimens and treated demineralized specimens. The esthetic results were significantly better in this group than in the microabrasion and nHAP groups. In contrast to our study, nHAP application significantly improved the color of WSLs. Both the microabrasion and nHAP groups showed a significant difference to the control group, with no differences between them. The inconsistency in the results may be due to the differences in nano-hydroxyapatite formulations and mode of application. In the study by Hammad et al., the specimens were brushed with nHAP toothpaste for 60 s two times per day for ten consecutive days. Our study implied a single application of a professional paste for 30 s that could be insufficient to induce significant color improvement.

The camouflage effects of resin infiltration and microabrasion were also compared in a number of studies [21,35,47]. In the study by Yetkiner, only the infiltration treatment improved the whitish appearance of the lesions back to their baseline levels [47]. Microabrasion also significantly decreased the L* values, but the lightness parameter was still significantly higher than the baseline readings. In a randomized clinical study by Gu et al., ΔE decreased significantly after treatment in both groups, with no differences between them [21]. A recent systematic review comparing infiltration, microabrasion, and various remineralizing agents concluded that infiltration provided the most effective and predictable esthetic result of WSL treatment [10].

Our study focused on the comparison of short-term aesthetic outcomes of remineralization, resin infiltration, and microabrasion, while long-term effects of the methods were not assessed due to the in vitro nature of the study. Previous clinical studies have shown a sufficient durability of resin infiltration for 6 [35], 12 [55,56], 24 months [57], and 6 years [56]. Interestingly, in one study, a continuous esthetic improvement was reported within the first 3 months of follow-up [58]. In the study by Gu et al., both resin infiltration and microabrasion showed sufficient durability for 12 months; however, the former method showed a better esthetic improvement at 12 months of follow-up [21]. According to Shan et al., resin infiltration and microabrasion were similarly effective in decreasing the WSL sizes, but resin infiltration offered an esthetic advantage over microabrasion [59].

In our study, we used coffee as a staining solution, as it was found to cause the most intense and dark staining among different discoloring agents [41,60]. A 7-day storage time was chosen as it is approximately equivalent to 3 years of daily consumption of two cups of coffee (5 min per each cup). Previous studies utilized highly variable discoloration protocols: the time of immersion varied from 8 h to 28 days; some of the authors used intermittent discoloration procedures (i.e., 10 min daily for 70 [61] or 50 days [62] equaling 12 and 8 h in total, respectively). Some studies have shown that clinically unacceptable discoloration was found after 7 days or less [36,47,53,63].

The staining of the specimens treated with infiltration and microabrasion resulted in color changes (T2-T3) comparable to those of the sound enamel. These changes were smaller than in the other groups, although the differences did not reach the level of statistical significance. The most pronounced staining was found in the negative control group due to a decrease in lightness (L*) and increase in both of the chromaticity parameters (a* and b*). Specimens treated with nHAP showed a significant increase in the a* and b* parameters, while specimens treated with ICON and MA as well as sound enamel showed no significant changes in the L*, a*, and b* values. The specimens treated with resin infiltration were least affected by the discoloration procedure. Partially similar results were reported in the study by Yetkiner et al., where resistance to staining (black tea) was assessed after infiltration, microabrasion, and fluoride treatments of WSLs. The authors found a decrease in the L* value and an increase in the a* and b* values in all groups except for the infiltration group [47]. In contrast, Silva et al. concluded that resin-infiltrated enamel was more susceptible to color changes following immersion in coffee than enamel treated with microabrasion [63]. The controversy in the results may be explained by differences in the study designs, e.g., demineralization procedure, staining solution type, time of immersion, etc. [53]. A number of studies have assessed color stability of resin-infiltrated WSLs without comparisons with other treatment modalities. In a study by Sabti et al., the authors found notable changes in color lightness (ΔL) and color chromaticity (Δa and Δb) not only in the control group (intact enamel) but also in the infiltration group after immersion in coffee (compared to their baseline measurements). Pronounced staining in their study may be explained by a longer exposure period (30 days) [39]. Considerable staining in the infiltration group was also reported by Alqahtani et al. In this group, ∆E after a 7-day immersion in black coffee comprised 20.2 [36]. Limvisitsakul et al. found that resin-infiltrated specimens exposed to coffee for 7 days exhibited a considerable color change (∆E > 15) [53]. The controversy in the studies’ findings can be explained by the fact that in in vitro settings the results of color measurements are influenced by a variety of factors including specimen thickness, surface roughness, time of water storage, and aperture size [46]. Moreover, in a systematic review by Sacucci et al., it was concluded that the results of color stability testing differed considerably depending on the study design. While in vitro studies indicated that the infiltrated enamel was susceptible to staining by chromogenic agents, in vivo studies found no significant color changes in the long term [37]. A systematic review by Baptista-Sánchez et al. assessed color stability of resin-infiltrated WSLs after a 6-month follow-up. The authors reported considerable heterogeneity in the results and methodology, found a small number of eligible studies, and concluded that more long-term studies were necessary [64].

To the best of our knowledge, none of the studies evaluated color stability after nHAP application to the WSLs. In a study by Almulhim et al., the effect of another Ca-containing remineralizing agent (casein phosphopeptide amorphous calcium phosphate, CPP-ACP) on the color of WSLs was compared to the effect of resin infiltration. The authors found that coffee induced similar staining in the CPP-ACP and control groups that was significantly greater than that in the resin infiltration group [41]. However, the results are difficult to compare, as in our study we used a product with crystalline HAP that does not release calcium or phosphate ions and only fills the pores of the demineralized enamel.

The data from our study must be interpreted with caution because of the in vitro design of the experiment. An in vitro model fails to perfectly simulate the oral environment including salivary flow and buffer capacity, intake of various foods and beverages, and presence of dental deposits. Moreover, artificial WSLs may not completely imitate the complexity of natural enamel lesions. This study focused on the esthetic outcomes of WSL treatments and did not assess the outcomes related to structural enamel changes (e.g., microhardness and surface characteristics of the lesions). The use of a 7-day discoloration period might not fully represent the changes that occur in the WSLs over long periods. Further in vivo studies with longer follow-up periods, which will take these variables into account, are warranted.

In spite of its limitations, this study certainly adds to our understanding of the esthetic outcomes of WSLs treatments. The highest masking ability was achieved by resin infiltration and microabrasion; however, none of the treatment methods restored the original tooth color. Although the ∆E values of WSLs after infiltration and microabrasion were smaller than those in the nHAP and negative control groups, they were still higher than the clinically acceptable threshold. Infiltrated and microabraded lesions showed a tendency to better color stability than nHAP remineralized lesions.

## Figures and Tables

**Table 1:** Mean L* values (standard deviation) in the studied groups at different timepoints.

Timepoint	Positive Control (Sound Enamel)	Negative Control	nHAP	ICON	MA
T0	69.36 (2.50) ^Aa^	74.03 (2.20) ^Aa^	73.12 (4.32) ^Aa^	73.98 (4.82) ^Aa^	73.88 (5.00) ^Aa^
T1	69.09 (2.51) ^Ba^	79.53 (2.06) ^Cb^	81.22 (3.07) ^Cb^	83.03 (2.23) ^Cb^	80.48 (2.76) ^Cb^
T2	68.76 (2.92) ^Da^	81.55 (3.97) ^Eb^	80.98 (1.78) ^Eb^	67.08 (4.43) ^Dac^	70.34 (1.13) ^Dac^
T3	66.08 (2.90) ^Fa^	75.23 (1.97) ^Ga^	76.84 (2.74) ^Gab^	64.20 (3.23) ^Fc^	67.24 (1.07) ^Fc^

T0—baseline, T1—after demineralization procedure, T2—after treatment, T3—after discoloration procedure. ^ABCDEFG^ superscript uppercase letters indicate differences among the groups at each timepoint (rows); ^abc^ superscript lowercase letters indicate differences among different timepoints within the study group (columns).

**Table 2:** Mean a* values (standard deviation) in the studied groups at different timepoints.

Timepoint	Positive Control (Sound Enamel)	Negative Control	nHAP	ICON	MA
T0	−1.50 (1.63) ^Aa^	−1.03 (0.25) ^Aa^	−1.18 (0.26) ^Aa^	−1.03 (0.25) ^Aab^	−1.22 (0.46) ^Aa^
T1	−1.75 (0.97) ^Ba^	−2.00 (0.34) ^Bb^	−2.16 (0.19) ^Bb^	−2.18 (0.26) ^Ba^	−2.08 (0.04) ^Bb^
T2	−1.98 (0.72) ^Ca^	−1.93 (0.19) ^Cb^	−2.04 (0.23) ^Cb^	−1.50 (0.08) ^CDab^	−1.02 (0.41) ^Da^
T3	−0.72 (0.41) ^Ea^	−0.70 (0.34) ^Ea^	−1.20 (0.31) ^Ea^	−1.35 (0.51) ^Eb^	−0.68 (0.18) ^Ea^

T0—baseline, T1—after demineralization procedure, T2—after treatment, T3—after discoloration procedure. ^ABCDE^ superscript uppercase letters indicate differences among the groups at each timepoint (rows); ^ab^ superscript lowercase letters indicate differences among different timepoints within the study group (columns).

**Table 3:** Mean b* values (standard deviation) in the studied groups at different timepoints.

Timepoint	Positive Control (Sound Enamel)	Negative Control	nHAP	ICON	MA
T0	6.50 (2.65) ^Aa^	6.88 (1.61) ^Aa^	5.10 (0.71) ^Aa^	7.05 (1.49) ^Aa^	6.68 (1.87) ^Aa^
T1	5.96 (2.34) ^Ba^	−1.68 (1.05) ^Cb^	−2.52 (1.25) ^Cb^	−2.25 (1.01) ^Cb^	−2.62 (1.42) ^Cb^
T2	5.56 (2.52) ^Da^	−1.75 (0.82) ^Eb^	−3.64 (0.74) ^Eb^	4.88 (2.36) ^Da^	2.36 (1.71) ^Dc^
T3	6.55 (1.72) ^Ea^	6.00 (1.76) ^Ea^*	2.18 (1.01) ^Fc^	6.58 (2.59) ^Ea^	2.74 (1.11) ^Fc^*

T0—baseline, T1—after demineralization procedure, T2—after treatment, T3—after discoloration procedure. ^ABCDEF^ superscript uppercase letters indicate differences among the groups at each timepoint (rows); ^ab^ superscript lowercase letters indicate differences among different timepoints within the study group (columns); *the differences between the Control and MA groups are suggestively significant (*p* = 0.0628).

**Table 4:** Mean CIE-lab color difference values (standard deviation) in the studied groups between the study timepoints.

Group	∆E_T0-T1_	∆E_T0-T2_	∆E_T0-T3_	∆E_T1-T2_	∆E_T2-T3_
Positive control (sound enamel)	1.02 (0.59) ^a^	2.23 (1.05) ^a^	4.89 (2.37) ^a^	1.31 (0.61) ^a^	3.71 (2.26) ^a^
Negative control	10.23 (1.31) ^b^	11.71 (1.32) ^b^	3.14 (1.49) ^a^	9.00 (1.69) ^b^	10.08 (2.94) ^a^
nHAP	11.24 (2.05) ^b^	11.92 (1.83) ^b^	5.15 (1.67) ^ab^	6.52 (1.24) ^b^	7.26 (0.93) ^a^
Icon	13.47 (3.94) ^b^	7.46 (4.53) ^b^	10.46 (4.30) ^b^	20.98 (1.74) ^c^	4.13 (0.69) ^a^
MA	11.94 (4.08) ^b^	7.46 (2.48) ^b^	8.78 (3.40) ^ab^	14.40 (2.46) ^d^	3.49 (0.60) ^a^
*p*-value	<0.001	0.000242	0.0059	<0.001	0.0572

T0—baseline, T1—after demineralization procedure, T2—after treatment, T3—after discoloration procedure; ^abcd^ superscript letters indicate differences among the study groups (columns).

## Data Availability

The datasets used and/or analyzed during the current study are available from the corresponding author upon request.
